# 19q13.11 microdeletion concomitant with ins(2;19)(p25.3;q13.1q13.4)dn in a boy: potential role of *UBA2* in the associated phenotype

**DOI:** 10.1186/s13039-014-0061-z

**Published:** 2014-12-12

**Authors:** Carlos Venegas-Vega, Karem Nieto-Martínez, Alejandro Martínez-Herrera, Laura Gómez-Laguna, Jaime Berumen, Alicia Cervantes, Susana Kofman, Fernando Fernández-Ramírez

**Affiliations:** Unidad de Genética, Hospital General de México, Dr. Balmis 148, México, D.F 06726 México; Facultad de Medicina, Universidad Nacional Autónoma de México, Av. Universidad 3000, México, D.F 04510 México; Unidad de Medicina Genómica, Hospital General de México, Dr. Balmis 148, México, D.F 06726 México

**Keywords:** 19q13.11 microdeletion syndrome, Chromosomal rearrangement, *UBA2*

## Abstract

The 19q13.11 microdeletion syndrome (MIM613026) is a clinically recognisable condition in which a 324-kb minimal overlapping critical region has been recently described. However, genes not included within this region, such as *WTIP* and *UBA2*, have been proposed to contribute to the clinical characteristics observed in patients. Using cytogenetic techniques, single nucleotide polymorphism arrays, and the quantitative polymerase chain reaction, we identified a novel case with a 2.49-Mb deletion derived from a *de novo* chromosomal rearrangement. Based on a review of the literature, we support the notion that *UBA2* haploinsufficiency could contribute to the phenotype of this rare genomic disorder. UBA2 belongs to a protein complex with sumoylation activity, and several transcription factors, hormone receptors, and signalling proteins related to brain and sexual development are regulated by this post-translational modification. Additional clinical reports and further research on *UBA2* molecular function are warranted.

**Electronic supplementary material:**

The online version of this article (doi:10.1186/s13039-014-0061-z) contains supplementary material, which is available to authorized users.

## Background

The 19q13.11 microdeletion syndrome (MIM613026) is a clinically recognisable condition that has been recently identified by molecular karyotyping techniques. Only 11 cases have been reported, and the common clinical characteristics include intellectual disability, growth retardation, microcephaly, variable signs of ectodermal dysplasia, slender habitus, and genital malformations in males [1-7]. A minimal overlapping critical region (MOCR) of 324 kb has recently been identified ([hg18] chr19: 39,803,651-40,127,916) [4]; this MOCR includes four genes of the zinc finger family containing the Krüppel-associated box (KRAB domain) and two non-coding RNA (ncRNA) genes. Here, we report the first case of 19q13.11 microdeletion syndrome caused by a chromosomal rearrangement and discuss the potential role of *UBA2* in the phenotype of affected individuals.

## Case presentation

### Clinical description

**Figure 1:**
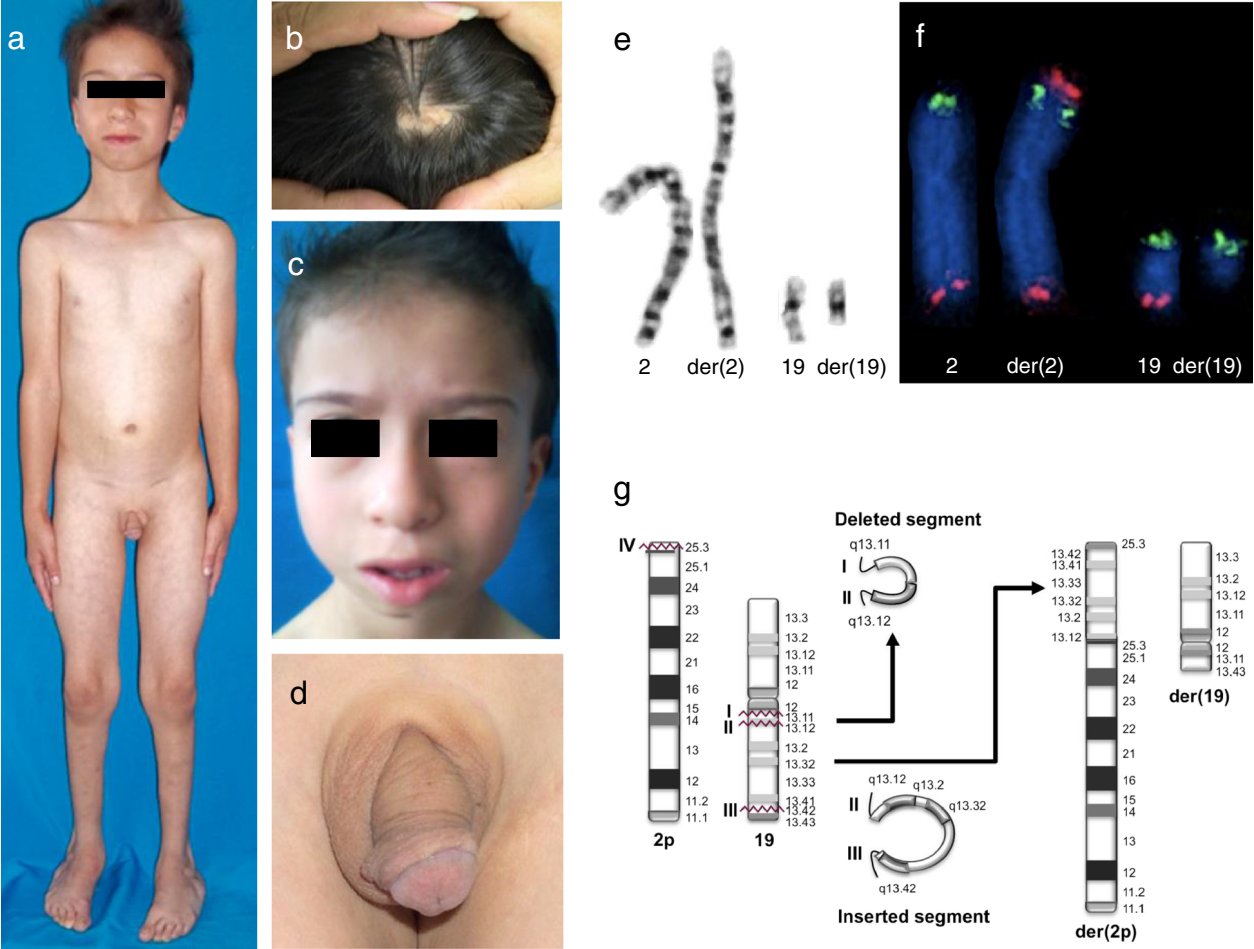
**Proband phenotype and cytogenetic analysis.** Proband at the age of 6 years and 7 months, showing **(a)** slender habitus with little subcutaneous fat and scars from the surgery for inguinal hernias; **(b)** cutis aplasia in midline scalp; **(c)** sparse hair, long face, high frontal hair line, sparse eyebrows and eyelashes, hypoplastic alae nasi, and low-set ears; and **(d)** shawl scrotum. **(e)** Partial GTG-banding karyotype of the patient showing normal and derivative chromosomes 2 and 19. **(f)** Normal and derivative chromosomes 2 and 19 showing FISH signals. Note the signal of 19q (orange) at the top of der(2), followed by the 2p (green) signal and the 2q (orange) signal at the end of the chromosome. der(19) shows only the 19p (green) signal. **(g)** Diagram illustrating the insertion of the segment from 19q13.12 to 19q13.43 in 2p25.3, with concomitant deletion of 19q13.11-q13.12.

The proband is the third child of non-consanguineous parents. Prior to his birth, the mother had one spontaneous abortion. Caesarean section was performed at 36.5 weeks of gestation because of preeclampsia. At birth, the patient showed low weight (<3rd centile) and length in the 10th–25th centile. The Apgar score was 7/10. Developmental delay, feeding difficulties, and recurrent upper airways infections compromised his early infancy. He underwent several surgical procedures because of bilateral hip dislocation, clubfeet varus, and hypospadias. At 5 years and 3 months of age, he had one febrile seizure, and 2 months later, he underwent surgery for bilateral inguinal hernia and left orchidopexy. Clinical evaluation was performed at 6 years and 7 months, and the weight was 16.1 kg (<3rd centile), the height was 112 cm (10–25 centile), and the occipital-frontal circumference was 46.5 cm (<3rd centile). The clinical findings are described in Figure 1a–d and Table 1. Hormonal studies, including analyses of FSH, LH, testosterone, oestradiol, progesterone, TSH, T3, T4, ACTH, cortisol, and growth hormone (basal and post-stimulation with glucose) all yielded normal results. Pelvic USG, EEG, audiometry and ECG yielded normal results.

**Table 1:** **Clinical features of patients with 19q13.11 deletion syndrome (MIM613026)**

**Patient**	**1**	**2**	**3**	**4**	**5**	**6**	**7**	**8**	**9**	**10**	**11**	**Proband**	**Total**
Size of the deletion (Mb)	11	6.16	4.27	3.19	2.4	1.74	2.63	7.87	1.37	8.16	2.30	2.49	
Gender	F	M	M	M	M	M	F	M	F	F	M	M	4 F/8 M
Age [years. months]	3.0	6.0	9.2	5.0	4.10	14	8.0	ϕ	6.5	5.6	1.6	6.7	
Preterm delivery [≤38 weeks]	+	+	+	+	+	+	+	+	+			+	10/10
***Development characteristics***													
Prenatal growth retardation	+	+	+	+	+	+	+	+	+	-	+	+	11/12
Feeding problems	+	+	+	+	+	+	-		+	+	+	+	10/11
Postnatal growth retardation	+	+	+	+	+	+	+		+	+	+	+	11/11
Slender habitus	+	+	+	+	-	+	-		+			+	7/9
Little subcutaneous fat	+	+	+	+			-		+			+	6/7
DD/ID	+	+	+	+	+	+	+		+	+	+	+	11/11
Speech disturbance	+	+	+	+	+	+	+		+	+	+	+	11/11
Microcephaly	+	+	+	+	+	+	+		+	+	+	+	11/11
Minor Facial dysmorphic features*	+	+	+	+	+	+	+	+	+	+	+	+	12/12
Long face	+	+	+	+	+	+	**-** ^a^	+	+	+		+	10/11
High frontal hairline	+	+	+	+^b^	+	+		+	+	+		+	10/10
High forehead	+	+	+	+	+	+			+	+		+	9/9
Eye abnormalities	+^c^	-	+^d^	+^e^	-		+^f^					+^c^	5/7
V-shaped nasal tip	+	+	+	+	+	+				+		+	8/8
Hypoplastic nasal alae	+	+	+	+	+	+				+		+	8/8
Low-set columella		+	+			+	+					+	5/5
Thin lips	+	+	+	+	+	+	+	+		+		+	10/10
Retro-micrognathia	+	+	+	+	+	+	+	+	+	-	+	+	11/12
Large ears or low-set ears	+	+	+	+	+	+	+	+	+	+		+	11/11
***Signs of ectodermal dysplasia***													
Aplasia cutis in midline of scalp	+	+	+	+	+	+	**-**		+	-	+	+	9/11
Thin/dry skin		+	+	+	+				+	-	-	+	6/8
Thin-sparse hair		+	+	+	+	+	**-**		+	-	-	+	7/10
Thin-sparse eyebrows/eyelashes	+	+	+	+	+	+	+		+	-	-	+	9/11
Teeth abnormalities		+^g^			+^h^	-	+^i^			+^j^	-	+^j^	5/7
Dysplasic nails		+	+	-	+	-	-		+	-	-	+	5/10
***Genital abnormalities***													
Hypospadias	NA	+	+	+	+	+	NA	+	NA	NA	+	+	8/8
Testicular ectopia	NA	-	+	+	+	-	NA		NA	NA	+	+	5/7
Bifid scrotum	NA	+	-	-	+	-	NA		NA	NA	-	+	3/7
***Extremity abnormalities***													
Long/tapering fingers	+	+	+		+	+	+			+		+	8/8
Clinodactyly of the 5th finger	+	+	+	+	+	+	-					+	7/9
Abnormal positioning of the feet	+				+	+			-			+	4/5
Overlapping of the toes	+	+	-	-	-	-	-		+			+	4/9
Cutaneous syndactyly F/T		-	+	+	+	+	-		-	+	+	+	7/10
***Others***													
Recurrent airways infections	+			+	+	+	-		+			+	6/7
Heart disease	+		+		-	+	-		+	-	+	-	5/9
Inguinal hernia				+							+	+	3/3
Febrile seizure									+		+	+	3/3
Endocrine abnormalities		+				+			+			-	3/4

Patients: **(1)** Kulhayra *et al*, [1] **(2–4)** Malan *et al*, [2] **(5)** Schuurs-Hoeijmakers *et al*, [3] **(6–7)** Gana *et al*, [4] **(8)** Lin *et al*, [5] **(9)** Forzano *et al*, [6] and **(10–11)** Chowdhury *et al*. [7] The total number of patients with a specific phenotype differs depending on whether the phenotype was specifically mentioned in the reports; only those reported are counted, and blank spaces correspond to data not documented. *We included the reported facial features and also features that were not reported in cases where evaluation of the published photographs was possible. *Abbreviations*: F, female; M, male; ϕ, foetus aborted at the 28th week of gestation; DD/ID, developmental delay/intellectual disability; F/T, fingers or toes; +, present; -, absent; NA, not applicable. Clinical findings: ^**(a)**^round face, ^**(b)**^frontal upsweep of hair, ^**(c)**^strabismus, ^**(d)**^microcornea-cataract, ^**(e)**^epiblepharon, ^**(f)**^astigmatism, ^**(g)**^single median incisor, ^**(h)**^teeth irregularly placed, ^**(i)**^hypodontia and ^**(j)**^multiple caries.

### Results

Karyotyping revealed a *de novo* rearrangement between chromosomes 2p25.3 and 19q13.1 (Figure 1e). FISH analysis with subtelomeric probes showed a signal of 19q on der(2), which retained the signal of 2p and 2q, whereas der(19) presented only the 19p signal (Figure 1f). The patient’s chromosomal complement was 46,XY,ins(2;19)(p25.3;q13.1q13.4)dn.ish ins(2;19)(p25.3;q13.1q13.4)(D19S238E+,U32389+,D2S447+;129F16/SP6+,D19S238E-). Microarray analysis indicated a 2.49-Mb *de novo* deletion (arr[hg19] 19q13.11-q13.12 (33,565,628–36,055,467) × 1 dn), which was confirmed by quantitative PCR (see Additional file 1: Figure S1). Trio SNP analysis of chromosome 19q revealed that the deleted allele was paternal, as indicated by 13 informative markers within the deletion (p < 1 × 10^-30^) (Additional file 2: Table S1). Taken together, these data indicate that the patient’s rearrangement corresponds to an insertion coupled with an interstitial deletion. His final chromosomal complement was 46,XY,ins(2;19)(p25.3;q13.12q13.43),del(19)(q13.11q13.12)dn.

### Discussion and conclusions

Eleven cases of 19q13.11 microdeletion syndrome have been reported [1-7], and two additional cases are annotated in the DECIPHER database (patients 127 and 3776) [8]. The parental origin of the reported 19q13.11 deletions suggests that an imprinting effect associated with this region is unlikely, because two maternal [3,4] and two paternal cases have been documented [1], including the one reported here. To our knowledge, this is the first case of a 19q13 deletion derived from a chromosomal rearrangement, which probably involved three breakpoints in chromosome 19 (at q13.11, q13.12, and q13.4) and one breakpoint in 2p25.3 (Figure 1g).

**Figure 2:**
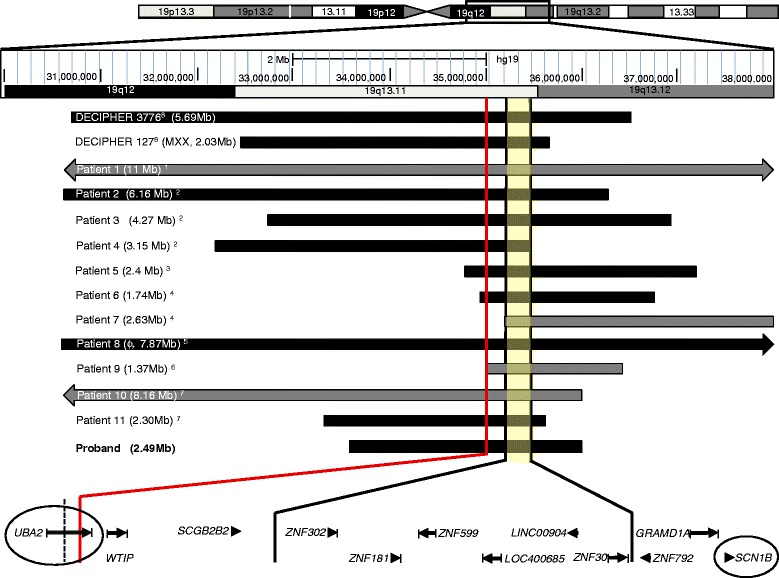
**Schematic summary of the reported 19q13.11 microdeletions, according to the human genome assembly hg19 (GRCh37).** An ideogram of Chr. 19q12-q13.12 is displayed at the top. The shaded region between the solid black lines represents the MOCR of approximately 324 kb ([hg19] chr19:35,111,811-35,436,076). The solid red line at the left indicates the deletion breakpoint of the patient reported by Forzano *et al*. ([hg19] chr19:34,957,764-34,983,674) [6], which affects the last two exons of *UBA2*. The proximal breakpoint of the male patient reported by Gana *et al.* [4] ([hg18]chr19:39,608,712-39,626,575) is indicated by a short dotted line; this deletion affects the last 11 exons of *UBA2*. The gene map is displayed at the bottom, and qPCR validation targets are circled. Patients are numbered according to Table 1, and deletions are indicated with grey (female) and black (male) bars; the size is indicated in parentheses. MXX is a 46,XX male; ϕ, male foetus aborted at the 28th week of gestation.

This patient displayed the main clinical features of 19q13.11 microdeletion syndrome (Table 1), and his deletion affected 49 genes, including those at the MOCR (Figure 2). Among these genes, *ZNF302*, *ZNF181*, *ZNF599*, and *ZNF30* belong to the KRAB-containing zinc finger subfamily and have been described as ubiquitous transcription repressors [9], whereas the ncRNA genes *LOC400685* and *LINC00904* are still uncharacterised.

Excluding one case involving a female individual (Patient 7, Table 1) [4], all of the reported deletions affected three genes in addition to those at the 324 kb MOCR: *UBA2, WTIP*, and *SCGB2B2* (Figure 2). SCGB2B2 is a member of the secretoglobin protein family whose function is unknown to date. These proteins are found at high concentrations in various human fluids and have been recently found to play immunomodulatory roles [10]. The other genes, *UBA2* and *WTIP,* could contribute to several clinical characteristics. Analysis of their genomic and functional properties, such as overlapping with copy number variant (CNV) regions and haploinsufficiency (HI), may help to clarify their potential role in this syndrome. A prediction score for HI has been generated from highly significant differences in genomic, evolutionary, functional and network properties between 1,079 haplosufficient genes and 301 genes known to display HI [11]. In this model, ranks between 0% and 10% indicate that a gene is more likely to exhibit HI. For instance, *WTIP* has been proposed as a candidate gene associated with hypospadias [4,7]. This gene is located in a copy number variant (CNV) region (i.e. the deletion has been observed in >1% of healthy control samples) [12,13] and it is not likely to exhibit HI (57.1%). However, this gene should not be excluded as a candidate because of its functional characteristics [4]. The upstream gene *UBA2* has also been proposed as a candidate for this syndrome [3], and it was also deleted in all of the reported male patients (Figure 2). Interestingly, this gene is strongly predicted to display HI (2.5%) [11] and no deletion variant has been observed in its locus [13]. Moreover, functional interactions between WTIP and UBA2 are possible, as these proteins share common physical interactors (http://string-db.org) [14].

UBA2 participates in the sumoylation process as a subunit of the dimeric E1-activating enzyme. Sumoylation is a post-translational modification in which a small ubiquitin-like modifier (SUMO) protein is ligated to a target protein, affecting its structure, intracellular localisation, or activity. Several transcriptional regulators, hormone receptors, and cell signalling proteins are regulated in this manner [15]. For instance, the androgen receptor (AR) is negatively regulated by sumoylation at its synergy control (SC) motifs. This mechanism could be important for normal AR function, as suggested by the finding of a P390S mutation in the first SC motif of the AR in a paediatric patient with hypospadias (reviewed by Mukherjee *et al.* [16]). Therefore, we hypothesize that HI of *UBA2* could contribute to the genital abnormalities observed in male patients, either by an autonomous mechanism or by molecular interactions among the deleted genes. A female individual with a congenital hydroureter presented a 19q13.1 deletion not overlapping with the MOCR but still affecting *UBA2* [7]*.* This finding indicates that careful urogenital evaluation of female patients is also important. Other transcription factors relevant to sexual determination and differentiation, such as SOX9 and SF1, are regulated by sumoylation [17]. Therefore, it is possible that this post-translational modification could be particularly important in sexual development. Sumoylation is clearly emerging as a key determinant in the regulation of neuronal maturation and synapse formation and activity at different stages of brain development [18]. Hence, it is possible that HI of *UBA2* could also contribute to the intellectual disability phenotype of this syndrome.

## Conclusion

In conclusion, we report a novel case of 19q13.11 microdeletion syndrome caused by a chromosomal rearrangement and suggest that *UBA2* haploinsufficiency could contribute to the phenotypic outcome of the male patients. Additional clinical reports and future research on its molecular function will clarify its role in this syndrome.

### Methods

G-band karyotyping was performed according to standard protocols. The subtelomeric regions of chromosomes 2 and 19 were analysed by FISH using mixtures #2 and #14 from ToTelVysion multicolour DNA probes (Vysis Abbott Laboratories, Abbott Park, Illinois, USA) according to the procedure described by the supplier. Mix #2 contains probes for 2p (U32389, green), 2q (D2S447, orange), chromosome X centromeric region (aqua) and Xq/Yq subtelomeric region (green/orange). Mix #14 has only 19p (129F16/SP6, green) and 19q (D19S238E, orange) probes.

Initial copy number and genotyping analyses were performed on the trio using GeneChip Human Mapping Sty 250 K arrays (Affymetrix Inc., Santa Clara, CA, USA), and an additional set consisting of 30 Mexican mestizo controls was used as reference. This microarray contains probes corresponding to ~238,000 single nucleotide polymorphism (SNP) positions, which are distributed across the genome with a median inter-marker distance of approximately 5 kb. To refine the deletion breakpoints, the high-density Genome-Wide Human SNP 6.0 array was used for copy number analysis of the proband, and data from 30 control samples of the Mexican population obtained from the International HapMap3 project (www.hapmap.org) were used as the reference set. The SNP 6.0 array contains 1.8 million probes (from which 906,600 correspond to SNPs), with a median inter-marker distance of 680 bp. All of the microarray procedures were performed according to the manufacturer’s instructions. Genotype calls were generated with Genotyping Console 4.1 (Affymetrix Inc.), and copy-number analyses were performed using SNP & Variation Suite 7.5.6 (Golden Helix Inc., Bozeman, MT, USA). The human genome assembly used was GRCh 37/hg19 (Feb 2009).

The microarray findings were validated by quantitative PCR on samples of the trio and in-house controls. The Taqman assays were Hs02790577_cn and Hs02374215_cn, corresponding to the *UBA2* and *SCN1B* deleted genes, respectively. The assays were performed in a StepOne Plus instrument following the manufacturer’s protocol, and the *RPPH1* assay (catalogue #4403326) was included as the copy-number reference. Results were analysed using Copy Caller 2.0 software. All materials, instruments, and software used for qPCR analysis were from Life Technologies (Foster City, CA).

## Consent

Written informed consent was obtained from the parents of the patient for the publication of this case report. A copy of the written consent is available for review by the Editor-in-Chief of this journal.

## Additional files

Additional file 1: Figure S1 Trio quantitative PCR analyses confirmed the *de novo* deletion of the **(a)** *UBA2* and **(b)** *SCN1B* genes in the proband. The patient’s sample showed a delayed amplification curve relative to his parents and control samples, indicating a single copy of each gene. The amplification curve of each sample is according to the colour chart at the left, and target genes are indicated with arrows. The ribonuclease PRNA component H1 gene (*RPPH1*) was used as a diploid reference assay, and two different sets of pooled samples were included for comparison (sets A and B, 10 healthy controls each). Assays were performed in quadruplicate. Additional file 2
**Trio SNP analysis in chromosome 19q.**
