# Supplementary material for: 19q13.11 microdeletion concomitant with ins(2;19)(p25.3;q13.1q13.4)dn in a boy: potential role of UBA2 in the associated phenotype
Source: Mol Cytogenet. 2014 Dec 12;7:61. doi: 10.1186/s13039-014-0061-z (PMC4266984; doi:10.1186/s13039-014-0061-z)
Supplement: Additional file 1: Figure S1 — Trio quantitative PCR analyses confirmed the de novo deletion of the (a) UBA2 and (b) SCN1B genes in the proband. The patient’s sample showed a delayed amplification curve relative to his parents and control samples, indicating a single copy of each gene. The amplification curve of each sample is according to the colour chart at the left, and target genes are indicated with arrows. The ribonuclease PRNA component H1 gene (RPPH1) was used as a diploid reference assay, and two different sets of pooled samples were included for comparison (sets A and B, 10 healthy controls each). Assays were performed in quadruplicate. [file 13039_2014_61_MOESM1_ESM.pdf]

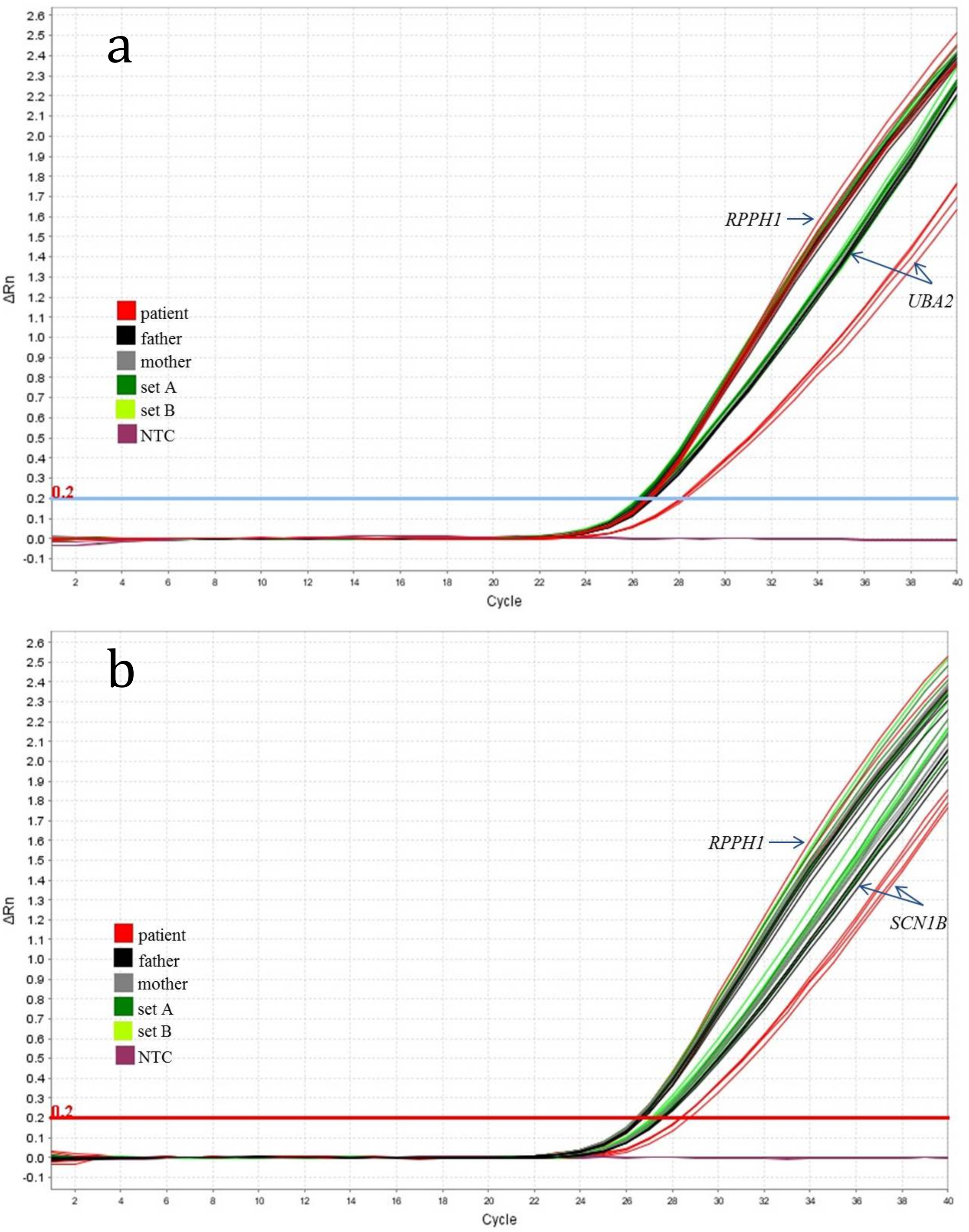

Additional Figure A1. Trio quantitative PCR analyses confirmed the *de novo* deletion of the (**a**) *UBA2* and (**b**) *SCN1B* genes in the proband. The patient's sample showed a delayed amplification curve relative to his parents and control samples, indicating a single copy of each gene. The amplification curve of each sample is according to the colour chart at the left, and target genes are indicated with arrows. The ribonuclease P-RNA component H1 gene (*RPPH1*) was used as a diploid reference assay, and two different sets of pooled samples were included for comparison (sets A and B, 10 healthy controls each). Assays were performed in quadruplicate.
